# Changes in suicidal ideation during treatment among patients with major depressive disorder: A 6‐month naturalistic follow‐up study

**DOI:** 10.1002/npr2.12428

**Published:** 2024-03-05

**Authors:** Aoi Sato, Norio Sugawara, Yasushi Kawamata, Norio Yasui‐Furukori

**Affiliations:** ^1^ Department of Psychiatry Dokkyo Medical University School of Medicine Mibu Japan

**Keywords:** major depressive disorder, naturalistic study, predictive factor, suicide ideation

## Abstract

**Aim:**

There is limited evidence regarding predictors of changes in suicidal ideation (SI) in patients with major depressive disorder (MDD). The objective of this study was to describe changes in SI over a 6‐month period and identify their predictors from naturalistic observations of MDD patients.

**Methods:**

In the cross‐sectional analysis, we examined 257 patients with MDD at the first‐visit assessment. Among the patients, 119 who completed the 6‐month assessment (completers) were included in the longitudinal analysis. For the evaluation of depressive symptoms, including SI, the Quick Inventory of Depressive Symptomatology‐Japanese version was administered at both the first‐visit and follow‐up assessments. At baseline, we also administered the Japanese version of the Ten Item Personality Inventory to assess personality traits and the PRIME Screen‐Revised to assess psychotic symptoms.

**Results:**

In the cross‐sectional analysis of first‐visit patients, 36.2% (93/257) exhibited SI. Among completers, 14.3% (17/119) had prolonged SI. Among the completers with SI at the first‐visit assessment, 38.6% (17/44) had SI at the follow‐up assessment (prolonged SI). In linear regression models including all completers, prolonged SI was positively associated with endorsement of suspiciousness/persecutory ideas and negatively associated with higher age.

**Conclusion:**

More than one‐third of completers who had SI at the first‐visit assessment experienced prolonged SI (SI at follow‐up). Our findings can help clinicians predict the course of MDD by identifying associated demographic and clinical characteristics.

## Abstract 2

A 6‐month naturalistic follow‐up study was conducted among patients with major depressive disorder. Among the completers with suicidal ideation at the first‐visit assessment, 38.6% (17/44) had it at the follow‐up assessment. Among all completers (*n* = 119), prolonged suicidal ideation was positively associated with suspiciousness/persecutory ideas and negatively associated with higher age.

## Graphical abstract

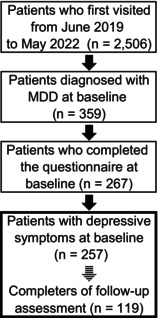

## 1 INTRODUCTION

Major depressive disorder (MDD) is a mental illness with a high prevalence,^1^ and patients have a 7.6‐fold higher overall suicide mortality rate than the general population.^2^ More than half of suicide victims previously had a mental illness, and 45% of all suicide victims had MDD.^3^ Currently, MDD is one of the leading contributors to the global burden of disease, accounting for 49.4 million disability‐adjusted life years worldwide.^4^ The disease burden of MDD may be increased by not only suicide completion but also suicidal behavior, including suicidal ideation (SI), suicide planning (SP), and suicide attempts (SAs).^5^ Suicidal behaviors are considered a strong predictor of completion of suicide,^6^ and a recent meta‐analysis estimated that 38% of MDD patients exhibit SI and 15% exhibit SP.^7^ As the prevalence of SI is high among patients with MDD, knowledge of the clinical course and predictors for SI is needed to improve MDD treatment.

However, as MDD patients with SI are often excluded from most clinical trials, limited data are available regarding their disease or treatment characteristics.^8^ Although there have been several studies on the clinical course of SI among patients with MDD,^9^, ^10^, ^11^ these studies have included patient samples, which are limited regarding specific severities of symptoms or exclude complex cases with physical comorbidities. Furthermore, interventions are controlled by protocols, which might not be the best practices, making it difficult to understand the real‐world features of MDD when using a controlled design. The abovementioned nature of controlled long‐term trials involving MDD patients could limit the generalizability of findings to broader clinical settings. In addition, clinical trials do not always focus on demographic characteristics or clinical heterogeneity.

There have been some naturalistic studies focusing on SI among patients with MDD.^12^, ^13^, ^14^ A cross‐sectional study from the US compared the characteristics of MDD patients with and without SI. In the analysis, MDD patients with SI were younger and had an earlier disease onset, a longer diagnosis latency, and a higher number of psychiatric comorbidities and depressive symptoms (e.g., feelings of hopelessness, impulsivity, self‐hating thoughts) than those without SI.^12^ Secondary analyses of cohort data from the Clinical Research Center for Depression (CRESCEND) study, in which participants consisted of MDD patients with clinically significant SI, revealed that 64% (359/565) of the participants still exhibited suicidality after 12 weeks of treatment, and higher levels of suicidality at baseline were associated with persistent suicidality after follow‐up.^13^ Another analysis of data from the same cohort showed that younger MDD patients had a lower rate of resolved suicidality than older patients at follow‐up.^14^ Although previous studies have clarified the naturalistic course of SI among patients with MDD, there have been few studies on how information about personality traits and symptoms at the first‐visit assessment can forecast subsequent changes in SI.

The objectives of this naturalistic study were as follows: (1) to evaluate the factors associated with SI at first‐visit assessment in a cross‐sectional analysis and (2) to identify predictive factors associated with prolonged SI after 6 months of follow‐up among patients with MDD.

## 2 METHODS

### 2.1 Design and participants

**FIGURE 1:**
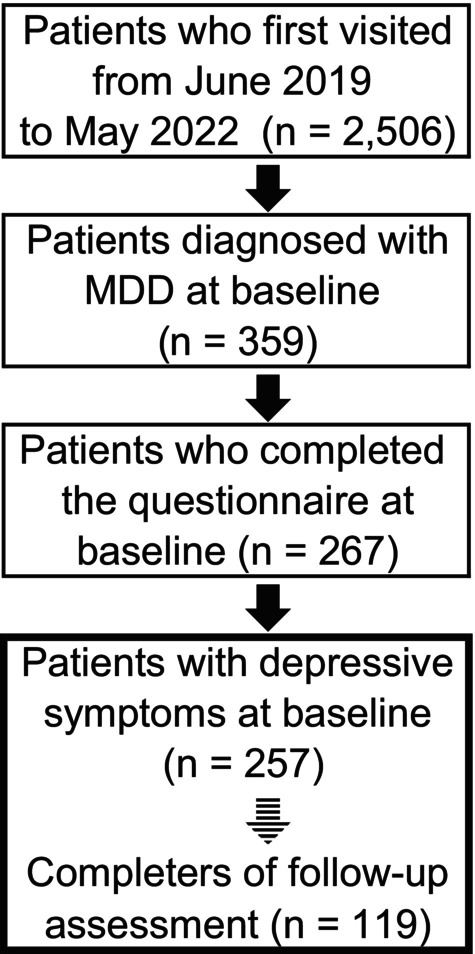
Flow chart of study sample selection. MDD, major depressive disorder.

This study was a 6‐month naturalistic follow‐up investigation of patients with MDD in real‐world clinical practice. We defined the first visit as the baseline and the assessment 6 months later as the follow‐up. First‐visit patients (*n* = 359) were diagnosed with MDD according to the Diagnostic and Statistical Manual of Mental Disorders, Fifth Edition (DSM‐5) between June 2019 and May 2022 at the Department of Psychiatry, Dokkyo Medical University Hospital. Among these MDD patients, 267 completed the baseline clinical assessment (the following questionnaire), and 257 had depressive symptoms according to the Japanese version of the Quick Inventory of Depressive Symptomatology (QIDS‐J). In this study, 6 months after the first visit, 119 patients with MDD completed the follow‐up assessment. The study included a cross‐sectional analysis of data from 257 patients at baseline and a longitudinal analysis of data from 119 patients (Figure 1). Data from patients with MDD were extracted from the Assessment for Identifying Subjective Symptoms in the Dokkyo Medical University Hospital, Psychiatric Service Use (AID‐P) database.^15^, ^16^ The AID‐P database, established at the Department of Psychiatry, Dokkyo Medical University Hospital in June 2019, gathers responses from clinical assessment questionnaires. This includes several questionnaires, such as the QIDS‐J. Upon their initial admission to the department, patients are required to complete these questionnaires. Thereafter, they are routinely assessed using these questionnaires at 6‐month intervals during their follow‐up period.

### 2.2 Measures

#### 2.2.1 Depressive symptoms including suicidal ideation

Self‐reported depressive symptoms were assessed with the QIDS‐J.^17^, ^18^ The QIDS‐J comprises 16 items and yields symptom ratings in nine domains. Responses are provided on a 4‐point Likert scale ranging from 0 to 3. Higher scores indicate more severe symptoms. Among the items, four (items 1, 2, 3, and 4) assess the sleep domain, and another four (items 6, 7, 8, and 9) assess the appetite/weight change domain. Furthermore, two items (items 15 and 16) are used to gauge psychomotor activity. For each of these three domains, the highest rating on any one relevant item is used to score the domain. Only one item is used to score the remaining six domains (feeling sad, concentration/decision‐making, view of myself, thoughts of death or suicide, general interest, and energy level). The maximum total score (sum of scores on the nine domains) is 27, and a QIDS‐J score of 6 or more is regarded as indicative of the presence of depression. In this study, thoughts of death or suicide were used as the outcome variable (SI). Based on the frequency, intensity, and specificity of the symptoms described by the response options statement, scores of 2 or 3 on thoughts of death or suicide were considered to endorse the symptom. Furthermore, we classified patients according to SI (presence of thoughts of death or suicide). Patients who did not exhibit SI at baseline or follow‐up were placed in the no SI group. Those with SI at baseline and without SI at follow‐up were placed in the “improvement in SI” group. In addition, those without SI at baseline and with SI at follow‐up were classified as the “emergence of SI” group. Patients with SI at both baseline and follow‐up were placed in the “prolongation of SI” group.

#### 2.2.2 Personality traits

The Ten Item Personality Inventory (TIPI) is a validated self‐report questionnaire in which the Big Five personality traits are assessed.^19^ The TIPI evaluates five distinct personality traits: extraversion, agreeableness, conscientiousness, emotional stability, and openness to experience. The TIPI consists of 10 items that are rated on a 7‐point Likert‐type scale. The average scores of the two items related to each trait are calculated to yield scores for each trait (ranging from 1 to 7), with higher scores indicating a higher level of the trait. In the Japanese version of the TIPI (TIPI‐J), “emotional stability” was translated as “shinkeisho keiko (neuroticism).” Note that “neuroticism” is the reverse score of “emotional stability”.^20^ In other words, the higher the “neuroticism” score in the TIPI‐J is the lower the emotional stability. The reliability and validity of the TIPI‐J have been confirmed.

#### 2.2.3 Psychotic symptoms

Psychotic symptoms over the past year were self‐reported on the Japanese version of the PRIME Screen‐Revised (PS‐R), which consists of 11 items that assess positive symptoms.^21^ To ensure the consistency of screening, the 12th item of the original PS‐R was excluded from the Japanese version because this item does not refer to attenuated positive symptoms. The 11 items reflect six categories of psychotic symptoms: perplexity and delusional mood (items 1 and 5), first‐rank symptoms (items 3, 6, and 11), overvalued beliefs (items 2 and 4), suspiciousness/persecutory ideas (item 7), grandiose ideas (item 8), and perceptual abnormalities (items 9 and 10). The PS‐R items are rated on a 7‐point Likert scale ranging from 0 (definitely disagree) to 6 (definitely agree). In this study, endorsement of each psychotic symptom category was evaluated according to a previous study.^5^ Items with scores of 5 (somewhat agree) or 6 (definitely agree) were counted as positive. The threshold for an endorsement of each psychotic symptom was one or more positive items on the symptom subscale.

### 2.3 Statistical analyses

Descriptive statistics of the demographic and clinical characteristics were calculated. To detect group differences, independent‐sample Student's *t* tests or one‐way analysis of variances (ANOVAs), as appropriate given the number of groups, were performed for continuous variables, and Chi‐square tests were performed for categorical variables. After ANOVA, a Tukey test was performed for post hoc comparisons. The data are presented as the means ± SDs or percentages.

To evaluate the factors associated with SI at baseline, multivariate logistic regression models with a forward selection method were constructed including the following independent variables: age, sex, TIPI‐J subscale scores, QIDS‐J domain scores (except for suicidal ideation), and PS‐R subscale categories. In the longitudinal analysis, two multivariate logistic regression models with a forward selection method were constructed that included the same independent variables to assess the factors predicting changes in SI. To characterize factors with prolonged SI during the follow‐up period, a multivariate logistic regression model with a forward selection method was constructed that included those who completed follow‐up (*n* = 119). Another model was employed to evaluate the factors related to prolonged SI among patients (*n* = 44) with SI at baseline (the “improvement in SI” group plus the “prolongation of SI” group). The threshold of statistical significance was set at 0.05, and analyses were conducted using SPSS for Windows, version 24 (IBM Corporation, Armonk, NY, USA).

### 2.4 Ethical considerations

The ethics committee of Dokkyo Medical University School of Medicine approved the protocol of this study (R‐78‐4J). Informed consent was obtained by providing opt‐out options on the website of the Department of Psychiatry, Dokkyo Medical University School of Medicine.

## 3 RESULTS

### 3.1 Cross‐sectional analysis of baseline data

The participants were divided into two groups according to SI status (present, *n* = 93; absent, *n* = 164). The clinical and demographic characteristics of the respondents are listed in Table 1. Age, TIPI‐J subscale (neuroticism) scores, some QIDS‐J domain (feeling sad, concentration/decision‐making, self‐esteem, thoughts of death or suicide, general interest, and energy level) scores, and PS‐R subscale (first‐rank symptoms) scores were significantly different between the two groups. In a multivariate logistic regression model with a forward selection method (Table 2), younger age and higher scores on some QIDS‐J domains (feeling sad and energy level) were significantly related to the presence of SI at baseline.

**TABLE 1:** Demographic and clinical characteristics of first‐visit patients.

	Suicidal ideation at baseline						
	Present		Absent		*p* Value	All patients	
	*n* = 93		*n* = 164			*n* = 257	
Age	39.2 ± 17.2		51.1 ± 18.6		<0.001	46.8 ± 18.9	
TIPI‐J scores							
Extraversion	3.6 ± 1.6		3.8 ± 1.5		0.255	3.7 ± 1.5	
Agreeableness	5.0 ± 1.2		5.2 ± 1.0		0.351	5.1 ± 1.1	
Conscientiousness	3.8 ± 1.5		4.1 ± 1.4		0.122	4.0 ± 1.5	
Neuroticism	5.4 ± 1.4		4.9 ± 1.1		0.013	5.1 ± 1.2	
Openness to experience	3.6 ± 1.4		3.7 ± 1.2		0.590	3.6 ± 1.3	
QIDS‐J scores							
Sleep	2.6 ± 0.7		2.4 ± 0.7		0.155	2.5 ± 0.7	
Feeling sad	1.9 ± 0.9		1.4 ± 0.9		<0.001	1.6 ± 0.9	
Appetite/weight change	1.9 ± 0.9		1.9 ± 1.1		0.948	1.9 ± 1.0	
Concentration/decision‐making	1.8 ± 0.9		1.5 ± 0.9		0.017	1.6 ± 0.9	
View of myself	2.0 ± 0.8		1.7 ± 0.8		0.003	1.8 ± 0.8	
Thoughts of death or suicide	2.3 ± 0.5		0.7 ± 0.5		<0.001	1.3 ± 0.9	
General interest	2.2 ± 0.9		1.8 ± 1.0		0.003	2.0 ± 1.0	
Energy level	2.0 ± 0.8		1.7 ± 0.8		0.006	1.8 ± 0.8	
Psychomotor	1.6 ± 0.9		1.4 ± 0.9		0.061	1.5 ± 0.9	
					**Chi‐square test**		
Male sex	30.1	(28/93)	40.2	(66/257)	0.105	36.6	(94/257)
PS‐R categories							
Perplexity and delusional mood	16.9	(15/89)	10.6	(17/160)	0.159	12.9	(32/249)
First‐rank symptoms	27.0	(24/89)	13.8	(22/160)	0.010	18.5	(46/249)
Overvalued beliefs	3.4	(3/89)	3.1	(5/160)	0.916	3.2	(8/249)
Suspiciousness/persecutory ideas	10.1	(9/89)	5.0	(8/160)	0.125	6.6	(17/249)
Grandiose ideas	0.0	(0/89)	0.0	(0/160)		0.0	(0/249)
Perceptual abnormalities	10.1	(9/89)	5.6	(9/160)	0.190	6.6	(18/249)

Abbreviations: PS‐R, PRIME Screen‐Revised; QIDS‐J, Quick Inventory of Depressive Symptomatology‐Japanese version; TIPI‐J, Japanese version of the Ten Item Personality Inventory.

**TABLE 2:** Predictors of suicidal ideation among first‐visit patients (*n* = 257).

	Odds ratio	95% CI	Wald value	*p* Value
Age	0.96	0.95–0.98	18.83	<0.001
QIDS‐J score				
Feeling sad	1.59	1.15–2.20	8.00	0.005
Low energy level	1.80	1.22–2.65	8.88	0.003

*Note*: Multivariate logistic regression models with a forward selection method were performed with the presence of suicidal ideation as the dependent variable and age, sex, TIPI‐J subscale scores, QIDS‐J domain scores (except for suicidal ideation), and PS‐R categories as independent variables.

Abbreviations: PS‐R, PRIME Screen‐Revised; QIDS‐J, Quick Inventory of Depressive Symptomatology‐Japanese version; TIPI‐J, Japanese version of the Ten Item Personality Inventory.

### 3.2 Participant demographic and clinical characteristics at baseline

Overall, 46.3% (119/257) of participants completed the follow‐up assessment. Those who completed the follow‐up assessment were divided into four groups according to SI status (no SI, *n* = 70; emergence of SI, *n* = 5; improvement in SI, *n* = 27; and prolonged SI, *n* = 17). The clinical and demographic characteristics of the respondents are listed in Table 3. Age, TIPI‐J subscale (openness to experience) scores, some QIDS‐J domain (feeling sad, thoughts of death or suicide, and psychomotor activity) scores, and PS‐R subscale (first‐rank symptoms and suspiciousness/persecutory ideas) scores significantly differed among the groups.

**TABLE 3:** Demographic and clinical characteristics among completers of the follow‐up assessment according to changes in suicidal ideation.

	Changes in suicidal ideation during the 6‐month follow‐up period				*p* Value	Post hoc comparisons	All patients
	No suicidal ideation	Improvement in suicidal ideation	Emergence of suicidal ideation	Prolongation of suicidal ideation			
	*n* = 70	*n* = 27	*n* = 5	*n* = 17			*n* = 119
					**ANOVA**		
Age	48.4 ± 17.5	35.8 ± 17.5	43.0 ± 20.7	31.8 ± 12.9	<0.001	a, b	42.9 ± 18.2
TIPI‐J scores							
Extraversion	3.6 ± 1.3	4.2 ± 1.8	3.8 ± 2.4	3.1 ± 1.4	0.110		3.7 ± 1.5
Agreeableness	5.3 ± 0.9	5.0 ± 1.2	5.6 ± 1.3	5.1 ± 0.9	0.571		5.2 ± 1.0
Conscientiousness	4.2 ± 1.3	3.7 ± 1.3	3.6 ± 1.6	4.5 ± 1.4	0.235		4.1 ± 1.4
Neuroticism	5.0 ± 1.0	4.9 ± 1.5	5.3 ± 1.2	5.4 ± 1.5	0.586		5.0 ± 1.2
Openness to experience	3.4 ± 1.1	4.1 ± 1.3	3.7 ± 2.2	3.0 ± 1.5	0.038	e	3.5 ± 1.3
QIDS‐J scores							
Sleep	2.4 ± 0.8	2.6 ± 0.6	2.6 ± 0.5	2.6 ± 0.6	0.434		2.5 ± 0.7
Feeling sad	1.4 ± 0.8	1.9 ± 0.9	1.0 ± 0.7	1.9 ± 0.6	0.012	a (*p* = 0.076)	1.6 ± 0.8
Appetite/weight change	2.0 ± 1.0	2.0 ± 1.0	2.0 ± 1.2	1.6 ± 0.9	0.547		1.9 ± 1.0
Concentration/decision‐making	1.6 ± 0.8	1.9 ± 0.8	1.4 ± 0.5	1.8 ± 1.0	0.324		1.7 ± 0.9
View of myself	1.7 ± 0.8	2.0 ± 0.9	1.6 ± 1.1	2.1 ± 0.7	0.269		1.8 ± 0.8
Thoughts of death or suicide	0.7 ± 0.5	2.4 ± 0.5	0.6 ± 0.5	2.4 ± 0.5	<0.001	a, b, c, d	1.3 ± 0.9
General interest	1.9 ± 0.9	2.1 ± 1.0	2.0 ± 1.2	2.2 ± 0.8	0.569		2.0 ± 0.9
Energy level	1.8 ± 0.8	2.1 ± 0.8	1.8 ± 0.4	2.1 ± 0.6	0.188		1.9 ± 0.8
Psychomotor	1.4 ± 0.8	2.0 ± 0.9	2.0 ± 1.0	1.1 ± 0.6	0.004	a, d	1.5 ± 0.9
					**Chi‐square test**		
Male sex	44.3 (31/70)	29.6 (8/27)	60.0 (3/5)	41.2 (7/17)	0.476		41.2 (49/119)
PS‐R categories							
Perplexity and delusional mood	11.4 (8/70)	20.0 (5/25)	40.0 (2/5)	11.8 (2/17)	0.277		14.5 (17/117)
First‐rank symptoms	8.6 (6/70)	32.0 (8/25)	60.0 (3/5)	23.5 (4/17)	0.003		17.9 (21/117)
Overvalued beliefs	5.7 (4/70)	4.0 (1/25)	0.0 (0/5)	5.9 (1/17)	0.939		5.1 (6/117)
Suspiciousness/persecutory ideas	1.4 (1/70)	8.0 (2/25)	20.0 (2/5)	23.5 (4/17)	0.007		6.8 (8/117)
Grandiose ideas	0.0 (0/70)	0.0 (0/25)	0.0 (0/5)	0.0 (0/17)			0.0 (0/117)
Perceptual abnormalities	4.3 (3/70)	8.0 (2/25)	20.0 (1/5)	11.8 (2/17)	0.427		6.8 (8/117)

Abbreviations: ANOVA, analysis of variance; PS‐R, PRIME Screen‐Revised; QIDS‐J, Quick Inventory of Depressive Symptomatology‐Japanese version; TIPI‐J, Japanese version of the Ten Item Personality Inventory.

^a^ No suicidal ideation vs. improvement in suicidal ideation.

^b^ No suicidal ideation vs. prolongation of suicidal ideation.

^c^ Emergence of suicidal ideation vs. improvement in suicidal ideation.

^d^ Emergence of suicidal ideation vs. prolongation of suicidal ideation.

^e^ Prolongation of suicidal ideation vs. improvement in suicidal ideation.

Characteristics of completers and noncompleters by presence or absence of suicidal ideation at first visit are shown in Table S1. Among patients with SI at first visit, completers of follow‐up assessments were significantly younger than noncompleters. Although there were no other significant group differences, age, and TIPI‐J subscale (neuroticism and openness to experience) scores, approached significance between completers and noncompleters. There were no differences in follow‐up assessment completion rates according to whether or not the patient had SI at first visit (presence of SI; 47.3% [44/93], absence of SI 45.7% [75/164]).

### 3.3 Predictors of clinical outcomes according to linear regression models

Among all participants, prolonged SI was positively associated with the endorsement of suspiciousness/persecutory ideas and negatively associated with higher age (Table 4). Among patients with SI at baseline (Table 5), higher scores of openness to experience and psychomotor activity had a significant negative association with prolonged SI, while suspiciousness/persecutory ideas had a positive association with prolonged SI in a multivariate logistic regression model with a forward selection method.

**TABLE 4:** Predictors of prolongation of suicidal ideation among follow‐up completers (*n* = 119).

	Odds ratio	95% CI	Wald value	*p* Value
Age	0.97	0.93–1.00	4.80	0.028
PS‐R category				
Suspiciousness/persecutory ideas	7.44	1.57–35.16	6.41	0.011

*Note*: Multivariate logistic regression models with a forward selection method were performed with prolongation of suicidal ideation as the dependent variable and age, sex, TIPI‐J subscale scores, QIDS‐J domain scores (except for suicidal ideation), and PS‐R subscale categories as independent variables.

Abbreviations: PS‐R, PRIME Screen‐Revised; QIDS‐J, Quick Inventory of Depressive Symptomatology‐Japanese version; TIPI‐J, Japanese version of the Ten Item Personality Inventory.

**TABLE 5:** Predictors of the prolongation of suicidal ideation among follow‐up completers with suicidal ideation at baseline (*n* = 44).

	Odds ratio	95% CI	Wald value	*p* Value
TIPI‐J scores				
Openness to experience	0.32	0.12–0.87	4.96	0.026
QIDS‐J scores				
Psychomotor retardation	0.10	0.02–0.65	5.77	0.016
PS‐R category				
Suspiciousness/persecutory ideas	17.48	0.80–381.55	3.31	0.069

*Note*: Multivariate logistic regression models with a forward selection method were performed with prolongation of suicidal ideation as the dependent variable and age, sex, TIPI‐J subscale scores, QIDS‐J domain scores (except for suicidal ideation), and PS‐R subscale categories as independent variables.

Abbreviations: PS‐R, PRIME Screen‐Revised; QIDS‐J, Quick Inventory of Depressive Symptomatology‐Japanese version; TIPI‐J, Japanese version of the Ten Item Personality Inventory.

## 4 DISCUSSION

This study was conducted to investigate changes in SI and to identify predictors among patients with MDD. Among first‐visit patients, more than one‐third had SI and were younger, and some depressive symptoms (feeling sad and energy level) were associated with SI in the cross‐sectional analysis. Regarding completers of the follow‐up assessment, 14.3% had prolonged SI, and psychotic symptoms (suspiciousness/persecutory ideas) at the first‐visit assessment were associated with prolonged SI, while higher age protected against prolonged SI. Among the completers with SI at the first‐visit assessment, 38.6% had prolonged SI at the follow‐up assessment. Among the completers with SI at the first‐visit assessment, certain personality traits (openness to experience) and depressive symptoms (psychomotor activity) protected against prolonged SI, while certain psychotic symptoms (suspiciousness/persecutory ideas) were positively associated with prolonged SI.

Consistent with previous findings,^5^, ^14^, ^22^ our results showed that younger age was related to the presence of SI at the first visit and was a predictor of prolonged SI at follow‐up. Older individuals have more life experiences and, through exposure to challenging experiences, have developed more resilient coping strategies.^23^ These strategies might attenuate SI. In addition, younger individuals, who lack the breadth of life experiences of older individuals, might see their current circumstances as unchangeable and feel overwhelmed by the perceived pressures and uncertainties of the future.^24^ Nevertheless, being elderly is recognized as a risk factor for suicide among patients with MDD.^25^ It remains possible that older MDD patients do not express SI, not because they do not have it, but simply because they did not express it verbally.

In our results, higher scores of openness to experience were shown to protect against prolonged SI among completers with SI at the first‐visit assessment. While there have been several studies on personality traits and SI, they have reported conflicting results. In a cross‐sectional study comparing MDD patients with high and low levels of SI, patients with high levels of SI had lower scores of openness to experience.^26^ Another study comparing suicidal and nonsuicidal patients reported similar results.^27^ Individuals with high levels of openness to experience might respond positively to treatment, including psychotherapy, due to their ability to adapt to new experiences and ways of thinking. Furthermore, they might have the ability to develop positive visions of the future that promote recovery. However, some studies have demonstrated an inverse relationship between openness to experience and SI.^28^, ^29^ Openness to experience might facilitate the disclosure of SI. Additionally, a correlation between levels of openness to experience and SI was observed, particularly in contexts where social support was low.^30^ Under these circumstances, the candid articulation of SI could mitigate the risk of suicide, potentially by amplifying the attention and aid provided by social networks, enhancing treatment adherence, and facilitating clinical monitoring.

Our cross‐sectional findings showed that some depressive symptoms (feeling sad and low energy level) at the first visit had an association with SI, while another symptom (psychomotor retardation) predicted changes in SI at follow‐up. While previous cross‐sectional studies showing an association between several depressive symptoms and SI align with our results,^31^, ^32^, ^33^, ^34^ there have been few studies on how psychomotor symptoms can be indicative of future changes in SI. However, SI and psychomotor retardation are symptoms that tend to be alleviated first among patients treated for MDD.^35^ Severe psychomotor symptoms are easily recognized by others, which might increase the likelihood of early intervention by healthcare professionals and family members. Early intervention has been reported to be effective in ameliorating depressive symptoms.^36^ Furthermore, patients with more severe symptoms are also more likely to receive more intensive monitoring and treatment. It is possible that such intensive treatment might contribute to improvements in SI.

There have been several studies on the association between SI and psychotic symptoms.^37^ Suspiciousness or persecutory ideas could exacerbate cognitive distortions, leading to negatively skewed thoughts. Therefore, patients might perceive their environment and relationships as hostile or threatening, which can intensify feelings of hopelessness or lead to social withdrawal and isolation. Isolation is a well‐known risk factor for prolonged SI and suicide attempts, as it can exacerbate depressive symptoms and deprive the individual of social support. Furthermore, patients might avoid seeking help or fail to comply with treatment recommendations. This can result in untreated or undertreated depression, increasing the risk of prolonged SI. For managing psychotic symptoms in patients with MDD, antipsychotic medications are sometimes used.^38^ If Suspiciousness/persecutory ideas are contributing to SI in the patients, antipsychotic medications could potentially reduce the severity of SI. However, directly inferring that antipsychotic medication will improve suicidal ideation in these patients requires careful consideration. Each patient's treatment plan should be tailored to their specific needs and continuously evaluated for effectiveness and safety.

The current study had some limitations. First, this study was conducted at a single academic medical center at a specific geographic location in Japan. Thus, caution is merited regarding the generalizability of our findings. The setting of academic medical center might attract patients with certain severity levels of depression, possibly those with more severe cases seeking specialized care. In addition, patients at a single academic medical center might have different access to healthcare resources compared to the general population. This could influence the severity and course of depression observed. Second, several potential factors associated with SI, such as duration of illness, duration of untreated illness, adverse childhood experiences, household income, satisfaction with income, and physical illness, were not assessed in our study. Especially, our study lacked factors such as employment and social support, which are the focus of public health strategies to reduce SI.^39^ Third, this study focused on SI. Although several studies have reported that SI is closely linked to SAs or completed suicide, the current results cannot be generalized to severe forms of suicide behaviors such as SAs and completions. Fourth, higher dropout rates could affect our results. Among patients with SI at first visit, completers of follow‐up assessments were significantly younger than noncompleters. Negative association between higher age and prolonged SI might be affected by dropout of older patients with SI. Other factors, such as personality traits, also approach statistical significance and results should be interpreted with caution. Fifth, the possibility that some patients may have been transferred to other hospitals and died, cannot be ruled out, although We confirmed that there are no suicide completers in noncompleters of follow‐up assessment in the electronic medical record. Finally, the naturalistic setting of the study did not allow the evaluation of specific treatment regimens. Complementary data from experimental studies are needed.

In conclusion, more than one‐third of first‐visit patients had SI. Younger age and some depressive symptoms (feeling sad and low energy levels) were associated with depression in the cross‐sectional analysis. Furthermore, more than one‐third of completers with SI at the first‐visit assessment exhibited prolonged SI at follow‐up. Personality traits (openness to experience) and depressive symptoms (psychomotor retardation) protected against prolonged SI, while psychotic symptoms (suspiciousness/persecutory ideas) were positively associated with SI. This study is the first to determine how personality traits influence SI change in Japanese patients with MDD. Our findings can help clinicians predict the course of MDD by providing insights into associated demographic and clinical characteristics. Further studies are needed to disentangle the effects of other clinical and psychological factors on prolonged suicidality during MDD treatment.

## AUTHOR CONTRIBUTIONS

NS designed the study. AS obtained the data. NS performed the data analysis. AS, NS, YK, and NYF interpreted the data and wrote the manuscript. All the authors have read and approved the final manuscript.

## FUNDING INFORMATION

The authors did not receive any specific funding for this work.

## CONFLICT OF INTEREST STATEMENT

The authors declare that they have no competing interests. Norio Yasui‐Furukori is an Editorial Board member of Neuropsychopharmacology Reports and a co‐author of this article. To minimize bias, they were excluded from all editorial decision‐making related to the acceptance of this article for publication.

## ETHICS STATEMENT

Approval of the Research Protocol by an Institutional Reviewer Board: The institutional review board of Dokkyo Medical University School of Medicine approved the protocol of this study (approval number: R‐78‐4J).

Informed Consent: All participants agreed to provide informed consent online and confirmed their intention to participate in this survey.

Registry and registration no. of the study/trial: Not applicable.

Animal Studies: Not applicable.

## PATIENT CONSENT STATEMENT

Informed consent was obtained in the form of an opt‐out option on the website of the Department of Psychiatry, Dokkyo Medical University School of Medicine.

## Supporting information


Table S1.

## Data Availability

The institutional review board of Dokkyo Medical University School of Medicine has restricted data sharing because the data contain potentially identifying or sensitive patient information. Please contact the institutional review board of Dokkyo Medical University School of Medicine with data requests. Upon request, the board will decide whether to share the data.
